# Anxiety and depression among vaccinated anesthesia and intensive care doctors during COVID-19 pandemic in United Arab Emirates: a cross-sectional study

**DOI:** 10.1186/s43045-022-00179-z

**Published:** 2022-02-03

**Authors:** Dalia Nagui Rizk, Mohamed Abo Ghanima

**Affiliations:** 1 Department of Neuropsychiatry, Psychiatry Unit, Faculty of Medicine, Alexandria, Egypt; 2 Department of Anesthesia, NMC Hospital Sharjah, Sharjah, United Arab Emirates

**Keywords:** Anesthesia doctors, Intensive care doctors, Vaccination, Depression, Anxiety, COVID-19

## Abstract

**Background:**

The coronavirus disease 2019 (COVID-19) pandemic had an impact on frontline healthcare workers’ (HCW) mental health as they experienced depression, anxiety, and sleep disturbances. There is a need to investigate the impact on anesthesia and intensive care doctors (ICU), especially after the rise of vaccination. Anesthesia and ICU doctors are among the frontline HCW dealing with suspected and confirmed COVID-19 patients. Their job puts them at risk of developing psychological disorders because of the daily stress. The aim of the current study was to assess factors affecting anxiety and depression among vaccinated anesthesia and ICU doctors working in United Arab Emirates (UAE). A cross-sectional study targeting vaccinated anesthesia and ICU doctors in UAE was conducted during March 2021. Data were collected using an online questionnaire uploaded to Google Forms including two sections; the first section included question assessing personal data, professional background data, previous COVID-19 diagnosis, and type of vaccine received. The second section of the questionnaire included the Hospital Anxiety and Depression Scale (HADS). Logistic regression analysis was used to assess the association of different factors with anxiety and depression. Adjusted odds ratio (AOR) and 95% confidence intervals (CI) were calculated.

**Results:**

Significantly higher anxiety (46%) and depression (53.6%) were associated with the Pfizer–BioNTech vaccine. The lowest anxiety (23.9%) and depression (21.6%) scores were related to the Sinopharm vaccine. Anxiety was significantly higher for participants previously diagnosed with COVID-19 (AOR = 2.55), and depression was lower for those who deal with COVID-19-positive patients (AOR = 0.28). Anesthesiologists had significantly lower anxiety and depression than those specialized in both anesthesia and ICU (AOR = 0.32 and 0.51)

**Conclusions:**

Previous diagnosis with COVID-19, female gender, and medical comorbidities were associated with high rates of symptoms of anxiety and depression among anesthesia and ICU doctors. Regular monitoring of the mental health impact of COVID-19, especially after the availability of different vaccines, is recommended.

## Background

Coronavirus epidemic started in late 2019 in Wuhan, China, and soon afterward, the virus had spread globally and was then declared a pandemic by the World Health Organization [1]. Frontline healthcare workers (HCW) faced many challenges, including shortage of personal protective equipment, morbidity, mortality, uncertainty, work instability, and loss of colleagues and family members, resulting in high stress levels never experienced before [2].

The pandemic affected frontline HCW’s mental health as medical staff started to experience depression, anxiety, insomnia, grief, and symptoms of post-traumatic stress disorder. Since the beginning of the pandemic, research has examined the immediate as well as the long-term effects of the pandemic on frontline HCW’s mental health [3].

Research is increasingly focusing on the psychological impact of coronavirus disease 2019 (COVID-19) on HCW. Indeed, there is a need to investigate its impact on anesthesia and intensive care unit (ICU) doctors, especially after the start of the vaccination campaign. Anesthesia and ICU doctors are among the frontline HCW dealing with suspected and confirmed COVID-19 patients. They deal not only with the mild and moderate cases but also the critically ill ones with all COVID-19 complications. Their job puts them at risk of developing psychological disorders as a result of the daily stress. This stress originates from the risk of transmitting COVID-19 to family members and the risk of personally contracting it [4].

Frontline HCW were given priority in vaccine allocation in many countries due to the high-risk exposures to COVID-19. Research showed that being a HCW involved in managing COVID-19 patients is associated with vaccine acceptance [5, 6]. Willingness to take the COVID-19 vaccine by frontline HCW does not obviate their concerns about these vaccines; therefore, the assessment of COVID-19 vaccine impact on their psychological symptoms and mental wellbeing is an interesting point for research [7].

To the best of our knowledge, no published surveys specifically targeted anxiety and depression in relation to COVID-19 vaccination among anesthesia and ICU doctors.

### Aim

The aim of the study was to assess factors affecting anxiety and depression among COVID-19 vaccinated anesthesia and ICU doctors working in the United Arab Emirates (UAE).

## Methods

### Study design and setting

A cross-sectional comparative study targeting anesthesia and ICU doctors who were vaccinated in UAE was conducted. Data were collected using an online questionnaire during March 2021. Ethical approval was obtained from the Ethics Committee of the Faculty of Medicine, Alexandria University, with serial number 0304881. Informed consent was taken electronically before data collection.

### Participants and sampling

The sample size was based on assuming a 95% confidence level and a 5% margin of error. According to Magnavita et al. [8], 27.8% (95% CI = 18.52, 37.03) and 51.1% (95% CI = 40.78, 61.44) anesthesiologists suffered from anxiety and depression due to the COVID-19 pandemic. The confidence interval (CI) was calculated for anxiety and depression percentage to be 18.52%, 37.03% for anxiety and 40.78%, 61.44% for depression. The minimum sample size was calculated using MedCalc Statistical Software (MedCalc Software bv, Ostend, Belgium; https://www.medcalc.org; 2020) to be 89 participants. Anesthesia and ICU doctors working in UAE, who have received two doses of the COVID-19 vaccine were invited to participate. Unfortunately, it was not possible to have a control group from UAE as nearly all anesthesia and ICU doctors were vaccinated by this time.

### Data collection

The questionnaire was uploaded to Google Forms, and the link was sent to eligible participants through emails and WhatsApp groups of anesthesia and ICU doctors. Follow-up reminders were sent for potential participants to maximize the response rate [9]. No incentives were offered for the completion of the questionnaire. The questionnaire was preceded by a brief introduction about the study team, objectives, estimated time for completion, and details about the confidentiality of responses. The survey included two sections of closed-ended questions; the first section included personal data (age, gender, marital status, having children, smoking and alcohol consumption, previous psychiatric illness, and medical comorbidities) and professional background data (designation, specialty, experience years, and working with COVID-19 positive patients). Furthermore, it included two questions assessing previous COVID-19 diagnosis and types of administered vaccines. The second section of the questionnaire included the Hospital Anxiety and Depression Scale (HADS). The questionnaire was pilot tested on ten healthcare professionals to ensure the appropriateness of the questions, and their data were not included in the final analysis.

### Hospital Anxiety and Depression Scale (HADS)

This questionnaire consists of two subscales for anxiety and depression, each composed of 7 questions with a total of 14 questions [10, 11]. All items were scored on a 4-point Likert scale ranging from 0 (Not at all) to 3 (Most of the time). The total score for each subscale was the sum of the scores of the seven questions. This scale is used to classify participants into three categories: without anxiety and/or depression (scores from 0–7), possible anxiety and/or depression (scores from 8–10), and anxiety and/or depression (scores from 11–21) [12].

### Statistical analysis

Data were analyzed using the IBM SPSS software package for Windows version 20.0 **(**Armonk, NY: IBM Corp). Qualitative data were described using number and percentage. The Kolmogorov-Smirnov test was used to verify the normality of distribution. Quantitative data were described using range (minimum and maximum), mean, standard deviation (SD), median, and interquartile range (IQR). The dependent variables were anxiety and depression, while the independent variables were the personal and professional background variables. Logistic regression analysis was used to assess the association between different independent variables with anxiety and depression**.** Univariable logistic regression was performed including one independent variable at a time, followed by multivariable logistic regression including all independent variables in one model (adjusted model). Odds ratios (OR) and 95% confidence intervals (CI) were calculated. The significance of the obtained results was judged at the 5% level.

## Results

A total of 219 vaccinated participants were included. Table 1 represents the background information of the study participants. Most of the participants were males (69.4%), aged between 36 and 50 years old (63.9%), and married (82.6%). About 95% of the participants did not have any previous psychiatric illness, and most of them (75.3%) were previously diagnosed with COVID-19 and dealing with COVID-19-positive patients (91.8%).

**Table 1:** Sample characteristics (*n* = 219)

	***N*** (%)
**Gender**	
Male	152 (69.4%)
Female	67 (30.6%)
**Age (years)**	
20–35	49 (22.4%)
36–50	140 (63.9%)
51–65	30 (13.7%)
> 65	0 (0%)
**Marital status**	
Single	30 (13.7%)
Married	181 (82.6%)
Separated	8 (3.7%)
**Having children**	
No	47 (21.5%)
Yes	172 (78.5%)
**Designation**	
Specialist	131 (59.8%)
Consultant	74 (33.8%)
Resident	14 (6.4%)
**Years in profession**	
0–10	49 (22.4%)
11–20	130 (59.4%)
> 21	40 (18.3%)
**Specialty**	
Anesthesia	111 (50.7%)
Intensive care	34 (15.5%)
Both	74 (33.8%)
**Smoking**	38 (17.4%)
**Alcohol consumption**	26 (11.9%)
**Medical comorbidity**	**(*n*** **= 219)**
No	163 (74.4%)
Yes	56 (25.6%)
**Previous psychiatric illness**	
No	207 (94.5%)
Yes	6 (2.7%)
Maybe	6 (2.7%)
**Working with COVID-19 patients**	201 (91.8%)
**Previous COVID-19 diagnosis**	**(*n*** **= 219)**
No	165 (75.3%)
Yes	54 (24.7%)

*N* frequency, *%* percentage

Table 2 shows the prevalence of anxiety and depression among the study participants. About 51% and 44% of the participants suffered from anxiety and depression with mean ± SD scores = 6.8 ± 4.6 and 7.9 ± 4.5 for depression and anxiety, respectively.

**Table 2:** Reported anxiety and depression according to HADS scores (*n* = 219)

Total score anxiety	*N* (%)
Normal (0–7)	106 (48.4%)
Borderline /Abnormal (8–21)	113 (51.6%)
Median (Min.–Max.)	8 (0–19)
**Total score depression**	
Normal (0–7)	122 (55.7%)
Borderline /Abnormal (8–21)	97 (44.3%)
Median (Min.–Max.)	7 (0–18)

*Min* minimum, *Max* maximum

Table 3 represents anxiety and depression scores in relation to different vaccine types. Significantly higher anxiety (46%) and depression (53.6%) were associated with the Pfizer–BioNTech COVID-19 vaccine. The lowest anxiety (23.9%) and depression (21.6%) scores were related to Sinopharm vaccine.

**Table 3:** Relation between reported anxiety and depression and type of administered vaccine (*n* = 219)

Type of vaccine received	Total score anxiety		Total score depression	
	Normal (0–7) (***n*** = 106)	Abnormal (8–21) (***n*** = 113)	Normal (0–7) (***n*** = 122)	Abnormal (8–21) (***n*** = 97)
Sinopharm (*n* = 69)	42 (39.6%)	27 (23.9%)	48 (39.3%)	21 (21.6%)
AstraZeneca (*n* = 46)	12 (11.3%)	34 (30.1%)	22 (18%)	24 (24.7%)
Pfizer–BioNTech (*n* = 104)	52 (49.1%)	52 (46%)	52 (42.6%)	52 (53.6%)
***P***	0.001^*^		0.012^*^	

*χ*^2^ chi square test

*p*: *p* value for comparing between Total score anxiety, Total score depression, and type of vaccine received

^*^Statistically significant at *p* ≤ 0.05

Table 4 highlights the association between anxiety and different independent factors. Females, and those who were previously diagnosed with COVID-19, had significantly higher odds of anxiety than males (AOR = 1.95, 95% CI = 1.01, 4.02 and AOR = 2.55, 95% CI = 1.19, 5.47), while anesthesiologists had significantly lower anxiety than those who specialized in both anesthesia and ICU (AOR = 0.32, 95% CI = 016, 0.66). The unadjusted model shows that female participants, those aged 20–35 years and who were previously diagnosed with COVID-19, had significantly higher odds of anxiety (OR = 2.53, 95% CI = 1.38, 4.61, OR = 2.86, 95% CI = 1.11, 7.37, and OR = 2.85, 95% CI = 1.47, 5.51), while anesthesiologists had significantly lower anxiety odds than those specialized in both anesthesia and ICU (OR = 0.30, 95% CI = 0.16, 0.56).

**Table 4:** Logistic regression analysis for the association between different factors with anxiety symptoms (abnormal vs. normal)

		Unadjusted model		Adjusted model	
		***P*** value	OR (95%CI)	***P*** value	AOR (95%CI)
**Gender (females vs. males)**		0.003*	2.53 (1.38, 4.61)	0.04*	1.95 (1.01, 4.02)
**Age (years)**	20–35	0.03*	2.86 (1.11, 7.37)	0.23	2.16 (0.47, 9.95)
	36–50	0.92	0.96 (0.44, 2.12)	0.55	1.35 (0.51, 3.58)
	51+ ®	Reference category			
**Marital status**	Single	0.39	2.00 (0.42, 9.71)	0.33	3.21 (0.31, 3.47)
	Married	0.96	0.97 (0.24, 3.99)	0.38	3.44 (0.22, 5.75)
	Separated ®	Reference category			
**Having children (yes vs. no)**		0.12	0.59 (0.31, 1.15)	0.90	1.13 (0.18, 7.26)
**Designation**	Specialist	0.13	1.55 (0.88, 2.76)	0.97	0.97 (0.48, 2.03)
	Resident	0.06	3.28 (0.94, 11.42)	0.88	1.10 (0.33, 3.67)
	Consultant®	Reference category			
**Years in profession**	≤ 10 years	0.07	1.85 (0.96, 3.56)	0.88	1.10 (0.33, 3.67)
	> 10 years ®	Reference category			
**Specialty**	Anesthesia	< 0.001*	0.30 (0.16, 0.56)	0.002*	0.32 (0.16, 0.66)
	Intensive care	0.38	0.69 (0.30, 1.59)	0.995	1.00 (0.38, 2.62)
	Both ®	Reference category			
**Smoking (yes vs. no)**		0.89	1.05 (0.52, 2.12)	0.91	1.05 (0.45, 2.45)
**Alcohol consumption (yes vs. no)**		0.56	0.78 (0.34, 1.78)	0.08	0.38 (0.13, 1.11)
**Medical comorbidity (yes vs. no)**		0.12	1.64 (0.89,3.05)	0.07	1.99 (0.95, 4.19)
**Previous psychiatric illness (yes/maybe vs. no)**		0.29	1.94 (0.57, 6.65)	0.25	3.19 (0.45, 2.78)
**Working with COVID-19 patients (yes vs. no)**		0.53	1.37 (0.52, 3.61)	0.88	0.92 (0.31, 2.72)
**Previous COVID-19 diagnosis (yes vs. no)**		0.002*	2.85 (1.47, 5.51)	0.02*	2.55 (1.19, 5.47)

*OR* odds ratio, *AOR* adjusted odds ratio, *CI* confidence interval

*Statistically significant at *p* < 0.05

Table 5 shows the association between depression and different independent variables. Single participants had significantly lower depression than separated participants (AOR = 0.66, 95% = 0.07, 0.99). Also having children and alcohol consumption were associated with lower odds of depression (AOR = 0.46, 95% CI = 0.07, 0.39, and AOR = 0.21, 95% CI = 0.06, 0.75). Specialists had higher depression odds than consultants (AOR = 1.25, 95% CI = 1.35, 2.26), while anesthesiologists had lower depression odds than those who specialized in both anesthesia and ICU (AOR = 0.51, 95% CI = 0.24, 0.83). Having medical comorbidities was associated with significantly higher odds of depression (AOR = 6.47, 95% CI = 2.88, 5.49). On the other hand, those who deal with COVID-19-positive patients had significantly lower depression odds (AOR = 0.28, 95% CI = 0.09, 0.88).

**Table 5:** Logistic regression analysis for the association between different factors with depression symptoms (abnormal vs. normal)

		Unadjusted model		Adjusted model	
		***P*** value	OR (95%CI)	***P*** value	AOR (95%CI)
**Gender (females vs. males)**		0.17	0.66 (0.37, 1.19)	0.09	0.51 (0.23, 1.12)
**Age (years)**	20–35	0.34	1.56 (0.62, 3.92)	0.03*	5.73 (1.15, 8.59)
	36–50	0.77	1.13 (0.50, 2.51)	0.39	1.57 (0.57, 4.34)
	51+ ®	Reference category			
**Marital status**	Single	0.39	0.50 (0.10, 2.43)	0.03*	0.66 (0.07, 0.99)
	Married	0.82	0.85 (0.21, 3.49)	0.39	3.16 (0.23, 14.17)
	Separated ®	Reference category			
**Having children (yes vs. no)**		0.55	1.22 (0.64, 2.36)	0.04*	0.46 (0.07, 0.39)
**Designation**	Specialist	0.30	1.36 (0.76, 2.42)	0.002*	1.25 (1.35, 2.26)
	Resident	0.40	0.59 (0.17, 2.05)	0.22	0.34 (0.06, 1.89)
	Consultant®	Reference category			
**Years in profession**	≤ 10 years	0.82	0.93 (0.49, 1.76)	0.86	0.89 (0.24, 3.24)
	> 10 years ®	Reference category			
**Specialty**	Anesthesia	0.11	0.62 (0.34, 1.12)	0.02*	0.51 (0.24, 0.83)
	Intensive care	0.33	1.51 (0.66, 3.43)	0.17	0.45 (0.36, 1.68)
	Both ®	Reference category			
**Smoking (yes vs. no)**		0.26	1.50 (0.74, 3.03)	0.12	1.95 (0.84, 4.55)
**Alcohol consumption (yes vs. no)**		0.14	0.52 (0.22, 1.25)	0.02*	0.21 (0.06, 0.75)
**Medical comorbidity (yes vs. no)**		< 0.001*	3.72 (1.95, 7.10)	< 0.001*	6.47 (2.88, 5.49)
**Previous psychiatric illness (yes/maybe vs. no)**		0.68	1.28 (0.40, 4.08)	0.47	1.89 (0.34, 3.61)
**Working with COVID-19 patients (yes vs. no)**		0.32	0.61 (0.23, 1.61)	0.03*	0.28 (0.09, 0.88)
**Previous COVID-19 diagnosis (yes vs. no)**		0.51	1.23 (0.66, 2.28)	0.61	1.23 (0.56, 2.68)

*OR* odds ratio, *AOR* adjusted odds ratio, *CI* confidence interval

*Statistically significant at *p* < 0.05

## Discussion

To the best of our knowledge, this study is among the earliest to address the anxiety and depression symptoms among COVID-19 vaccinated anesthesia and ICU doctors. As many treatments are still currently under investigation for COVID-19 treatment [13], frontline HCW feel worried all the time. In addition, the emergence of different types of vaccines, even if they did not show full protection against the infection, might affect doctors’ psychological response.

In the current study, the prevalence of anxiety in the vaccinated group was 51.6%, and the prevalence of depression was 44.3%; on the other hand, anxiety prevalence was 60% and depression 61% in the non-vaccinated group. A study conducted by El Kholy et al. [14] in Egypt to evaluate mental health outcomes among HCW treating patients with confirmed or suspected COVID-19 in 2020, before the start of the vaccination program in Egypt, found the prevalence of anxiety was 77.3% and depression 79.3%. Other studies observed a prevalence of anxiety ranging from 23.2 to 32% and depression from 22.8 to 51.1% in anesthesia doctors, ICU doctors and other HCW [8, 15–17]. However, those studies used different screening tools, all done before vaccination, making the direct comparison of results difficult and showing the importance of measuring anxiety and depression post vaccination to compare results and check if vaccination helped in improving mental health. A UK-based study of ICU doctors conducted in 2018, before the COVID-19 pandemic, found that 16% of staff had significant anxiety and 8% had significant depression, showing the rise in anxiety and depression prevalence in this group of HCW [18].

According to the current study, higher anxiety and depression were significantly associated with the Pfizer vaccine, followed by the AstraZeneca vaccine. The lowest anxiety and depression were related to the Sinopharm vaccine. The new pioneering technology used to manufacture the RNA-based Pfizer vaccine and the adenovirus AstraZeneca vaccine has never been used in the manufacture of vaccines before. Hence many people had concerns about those vaccines’ efficacy in preventing the infection, their short-term side effects, and the unavailability of data on long-term effects, which might explain the higher association of anxiety and depression to those vaccines. Unfortunately, social media’s impact has been strong, spreading unconfirmed data about the emerging vaccines, which played a role in increasing the mass concerns, even among professionals. For example, a study conducted in Saudi Arabia showed that of all the HCW respondents asked about COVID-19 vaccines, only 20 or 24% preferred to receive the AstraZeneca or the Pfizer–BioNTech vaccine, respectively [19]. On the other hand, the Sinopharm vaccine was manufactured using the well-known standard technology, making it less anxiety-provoking to people.

We found that anxiety was significantly higher in females than males, confirming the results of Xiaoming et al. [20] and Flesia et al. [21] that showed higher levels of distress in females during the COVID-19 pandemic. Our findings were also conforming to the results by Magnavita et al. [8] who reported that the prevalence of distress in female anesthetists during the COVID-19 pandemic was 78.7% and that of anxiety was 29.8%, compared to 62.8% and 25.6%, respectively, in their male colleagues. Other studies also showed a gender difference in anxiety, demonstrating a higher level in females [22–24]. A factor that may contribute to the rise of anxiety among female doctors is the increase in their tasks and responsibilities at work and at home, resulting in excess in their workload. Furthermore, males are less likely to recognize or report psychiatric disorders [25, 26].

Anxiety was found to be higher among doctors who were previously diagnosed with COVID-19, showing the possible alarming impact of a previous COVID 19 infection on mental health as doctors have a continuous fear of re-infection. This was concordant with Mazza et al. [27] who reported a prevalence of 42% of anxiety in COVID-19 survivors. Frontline HCW who recovered from COVID-19 still struggled with their fear of re-infection as they deal with COVID-positive patients every day.

We found a statistically significant association between medical comorbidity and depression, as chronic health conditions may trigger depression. Lee et al. [28] examined the psychological distress during the COVID-19 pandemic among anesthesiologists and nurses working in ICUs in Singapore and found a significant association between the psychological distress and the presence of multiple comorbidities staff. In the current study, depression symptoms were significantly lower among single doctors; this indicates that single doctors might face less family-related work pressure making them more protected from depression. Our findings were similar to Zheng et al. [29] who showed that married anesthesiologists had poor mental health, and Zhu et al. [30] who reported that being single was a protective factor for depression in medical staff [31]. Furthermore, Doshi et al. [32] in India found that married people had a greater fear from COVID-19 than single people in the general population. On the other hand, Babor et al. [33] found no difference between single and married HCW regarding the perceived stress towards COVID-19.

Our results showed that depression symptoms were significantly lower in doctors who have children showing that the presence of children may bring a sense of hope that may protect against depression. Elbay et al. [34] conducted a study in Turkey and found that being married and having a child were associated with lower depression scores. In addition, several studies conducted during the COVID-19 pandemic found that HCW with children at home perceived less distress and focused more on positive aspects of their lives [35, 36]. Having children may help the doctors deal better with the exhausting working hours, intense workload, burnout, and frustration caused by the pandemic.

Contrary to our expectations, alcohol consumption was associated with lower depression symptoms. In our sample, around 10% were drinking alcohol, not a high enough prevalence to suggest that alcohol use is associated with lower depression possibility. High alcohol consumption can be seen in physicians, as shown by previous studies, with anesthetists having 2.7 times higher incidence than other specialties. This finding is different from Mikalauskas et al. [37], who observed an association between alcohol drinking and professional burnout in anesthesia and ICU physicians. Depression and burnout have been described as being overlapping and complementary phenomena [38–41].

Surprisingly, depression was found to be lower in doctors working with COVID-19-positive patients. Those findings were not in agreement with the findings published from Italy by Di Tella et al. [42], explaining that frontline HCW are in a daily struggle to keep the COVID-19 patients alive, have insufficient rest, are under a permanent threat of being infected, and are isolated from family due to their workload putting them at higher risk for depression. Our findings were contrary to the findings from Wuhan by Li et al. [43], who reported that anesthesiologists and nurses exposed to COVID-19 patients were more vulnerable to experience depression and anxiety. This study was performed during the period of Wuhan lockdown during the first COVID-19 wave before the availability of any vaccine and when little information was known about the virus. Our findings raised the question of whether vaccination might have given the frontline HCW a sense of safety and reassured them about being less infective to their families, which might explain the lower risk of depression. Another question is whether depression becomes less over time when dealing with COVID-19 patients due to more experience, awareness, and data availability about the virus giving the doctors a sense of familiarity. The answers of both questions need to be found through conducting more thorough research exploring vaccination effect on HCW mental health.

Overall, this study highlights the importance of properly protecting the mental health of anesthesia and ICU doctors facing COVID-19 patients considering the consequential impact on their ability to deliver high-quality patient care. Furthermore, it is considered a step for better understanding the effect of different types of COVID-19 vaccines on mental health as we had the opportunity to compare the results to a control group of doctors from the same two specialties who were not yet vaccinated. In addition, it could be useful to extend this research to other HCW in order to compare the results in different specialties.

## Conclusions

A previous diagnosis with COVID-19, female gender, and medical comorbidities were associated with high rates of symptoms of anxiety and depression among anesthesia and ICU doctors. Regular monitoring of the mental health impact of COVID-19, especially after the availability of different vaccines, is recommended.

### Limitations

A major limitation of the study was the lack of baseline data to allow for the comparison of the anxiety and depression symptoms before and during the COVID-19 pandemic, including before the availability of vaccination in the UAE. This was important to assess the temporal changes in anxiety and depression.

The study’s cross-sectional nature demonstrated an association between lower anxiety and depression and the COVID-19 vaccines, but it was difficult to prove causality.

Finally, the assessment depended on self-reporting, which might have introduced biases due to under-reporting and social desirability.

## Data Availability

The datasets used and/or analyzed during the current study are available from the corresponding author on reasonable request.

## Abbreviations

HCW: Health care workers
COVID-19: Coronavirus disease 2019
ICU: Intensive care unit
HADS: Hospital Anxiety and Depression Scale
SD: Standard deviation
IQR: Interquartile range
OR: Odds ratio
CI: Confidence interval
AOR: Adjusted odds ratio
